# Supplementary material for: SPAC: a scalable, integrated enterprise platform for end-to-end single cell spatial analysis of multiplexed tissue imaging
Source: bioRxiv. 2025 Apr 8:2025.04.02.646782. Preprint. [Version 1] doi: 10.1101/2025.04.02.646782 (PMC12026498; doi:10.1101/2025.04.02.646782)
Supplement: Supplement 1 — Supplementary Fig. 1 A representative Code Workbook on NIDAP illustrates the SPAC workflow from data aggregation and sampling, exploratory data analysis, feature normalization, clustering, dimensionality reduction, and phenotype annotation to spatial analysis in a unified, modular environment, ensuring consistent data lineage and minimizing format conversion burdens. Each analysis step produces downloadable .csv files and figures ready for scientific presentation. Integrated HPC Connector in PhenoGraph and UMAP modules enable seamless offloading of computations to GPU/CPU resources for efficient processing of large-scale datasets. An example analysis of normal lymph node tissue demonstrates NIDAP’s standardized yet flexible design, enabling bench scientists to configure parameters, launch workflows, and view results in real time without command line expertise, while data scientists can refine robust pipelines, with reproducibility and transparency maintained through version control, parameter tracking, and shared project workspaces. Supplementary Fig. 2 Hierarchical heatmap illustrating the expression profiles of key markers (columns) across PhenoGraph clusters (rows). Each cell represents the z-score of a given marker’s intensity (yellow= higher expression; purple=lower expression). The dendrograms show how clusters and markers group together based on similarity in expression patterns, revealing distinct subpopulations. Clusters 4 and 15, outlined in magenta boxes, exhibit high E-cadherin and β-catenin expression. Clusters 0 and 7, outlined in red boxes, display moderate PIMO expression. These highlighted clusters were subsequently merged and renamed in the final analysis (see Fig. 7). [file media-1.pptx]

## Slide 1
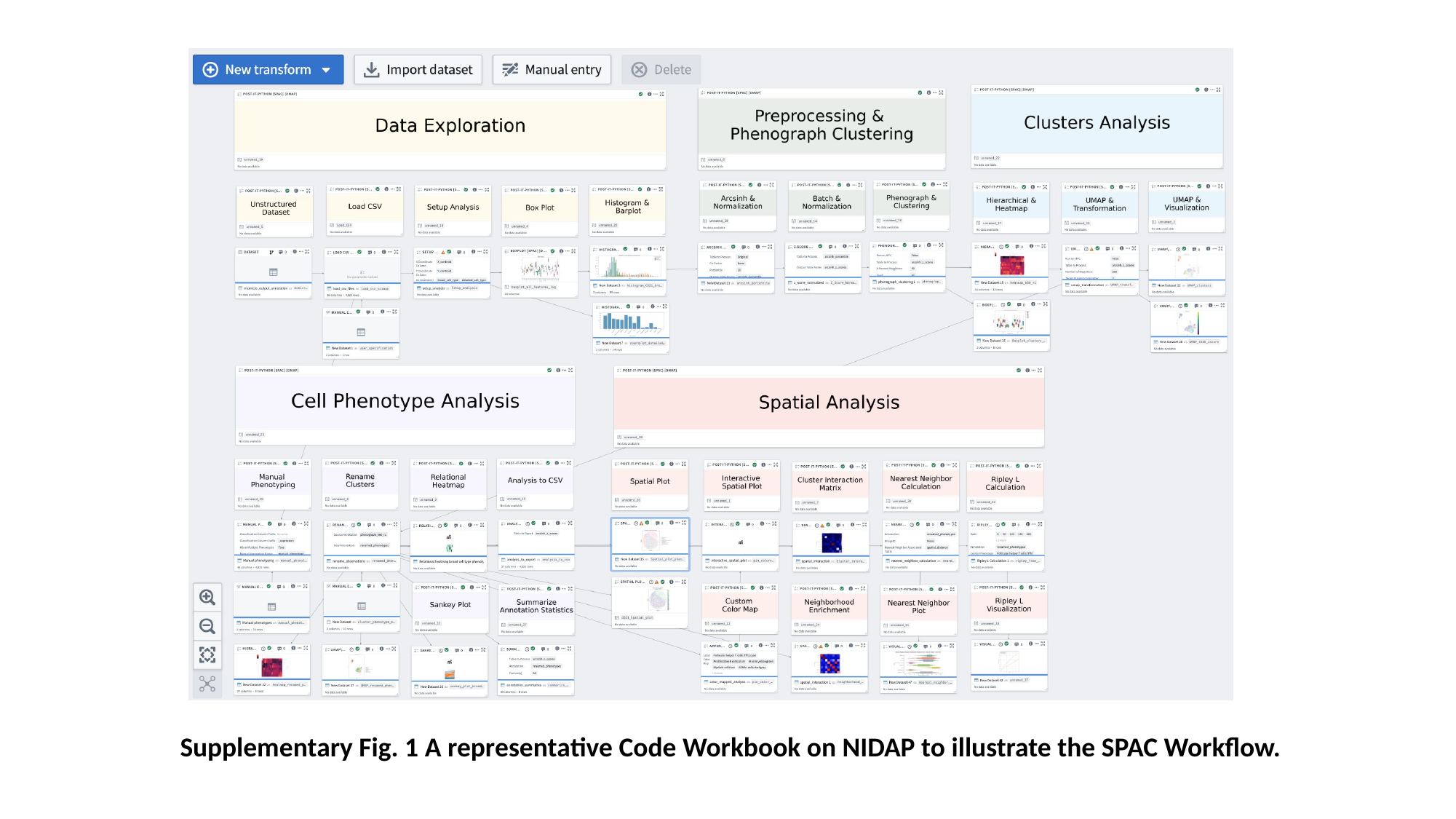

Supplementary Fig. 1 A representative Code Workbook on NIDAP to illustrate the SPAC Workflow.

## Slide 2
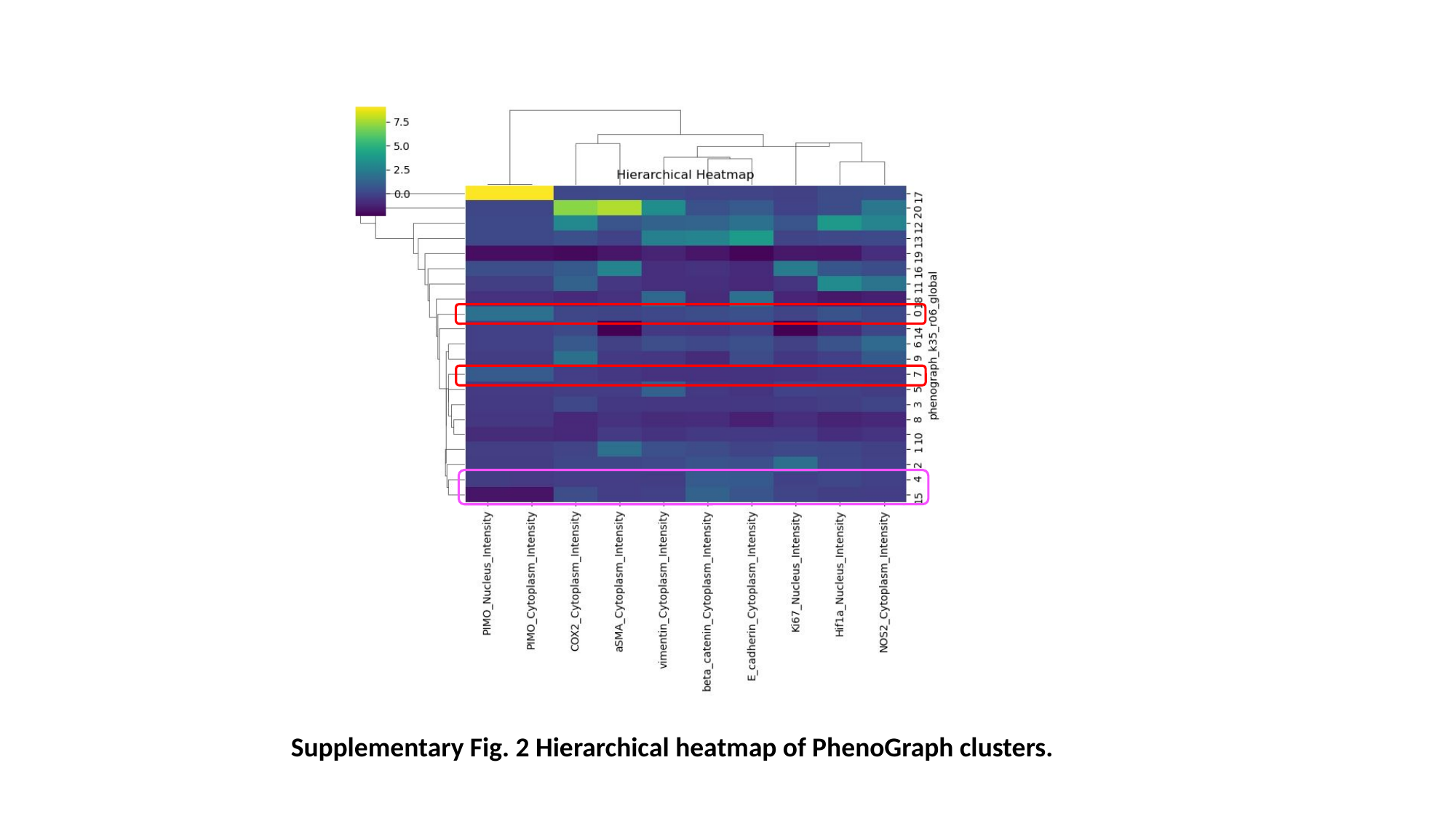

Supplementary Fig. 2 Hierarchical heatmap of PhenoGraph clusters.
